# Digital literacy and farmers’ entrepreneurial behavior—Empirical analysis based on CHFS2019 micro data

**DOI:** 10.1371/journal.pone.0288245

**Published:** 2023-07-26

**Authors:** Qingyun Bai, Haipeng Chen, Jie Zhou, Guohong Li, Dundang Zang, Yaya Sow, Qianling Shen

**Affiliations:** College of Economics, Sichuan Agricultural University, Chengdu, China; Alexandru Ioan Cuza University: Universitatea Alexandru Ioan Cuza, ROMANIA

## Abstract

Farmers’ entrepreneurship is an important engine to comprehensively help promote rural revitalization. Based on data from the China Household Finance Survey (CHFS2019), this paper empirically examines the effect of digital literacy on farmers’ entrepreneurial behavior using the Probit model and instrumental variable method; and examines the mediating role of health status in this effect using the mediation effect model, combined with the Sobel and Bootstrap tests. The results of the study showed that (1) digital literacy positively influenced farmers’ entrepreneurial behavior at the 1% significant level, and this positive influence showed some differences across gender and age; (2) health status played a mediating effect in the positive influence of digital literacy on farmers’ entrepreneurial behavior. Accordingly, policy recommendations are made to foster farmers’ digital literacy, encourage farmers’ entrepreneurship, and ensure farmers’ "digital health" services.

## Introduction

Rural revitalization strategy, as the overall grasp of the "San Nong" work in the new era, is a complex and huge systemic project. Fully mobilizing the enthusiasm and creativity of hundreds of millions of farmers and actively encouraging and supporting farmers’ entrepreneurship are essential measures to promote the rural revitalization strategy comprehensively [1], as well as to narrow the gap between urban and rural areas and enhance the new dynamic energy for the long-term stable development of China’s economy [2]. The "No. 1 Document" of the Central Government in 2019 proposed to "support rural innovation and entrepreneurship, encourage innovation and entrepreneurship among rural workers, and broaden the channels for farmers to increase their income" to guide farmers’ entrepreneurship and promote them their income correctly. However, the current lack of human capital and poor quality of access to information in rural areas [3] makes entrepreneurship’s survival rate generally low and entrepreneurship sustainability growth weak [4].With the promotion of digital village construction, the digital economy with the Internet, cloud computing, big data, artificial intelligence, and other carriers have been embedded in rural areas, giving rural areas a new development foundation and power mechanism [5]. Besides, it brought new business opportunities and market space and provided new opportunities for farmers to develop entrepreneurial activities. Digital literacy, as a "skill and asset in the digital economy," has also become one of the essential literacies farmers require, making entrepreneurial approaches and paths more diversified. The higher digitally literate farmers are, the more they have a significant influence on their entrepreneurial behavior through digital tools to broaden information channels, enhance social capital, improve digital capabilities [6], and stimulate innovation and entrepreneurial enthusiasm.

Totally can digital literacy promote farmers’ entrepreneurial behavior in real time? If the effect is confirmed, what is the mechanism of action behind it? How does the impact of digital literacy on farmers’ entrepreneurial behavior differ across individual characteristics? Existing studies still need to answer the answers to these questions clearly. Most of the existing studies on farmers’ entrepreneurial behavior have been analyzed in terms of essential individual traits [7], entrepreneurial environment [6–8], social capital [9], and infrastructure [10]. In contrast, there is a lack of research on the impact of digital literacy on farmers’ entrepreneurial behavior. An in-depth analysis of the influence of digital literacy on farmers’ entrepreneurial behavior and the correlation mechanism between them will help to more comprehensively understand the role of digital literacy in farmers’ entrepreneurial behavior, which is of great practical significance to accelerate the realization of rural revitalization. Therefore, this manuscript studies farmers’ entrepreneurial behavior from the perspective of digital literacy and is based on the survey data of the China Household Finance Survey (CHFS2019) to provide stage ideas for promoting farmers’ entrepreneurial behavior and realizing rural revitalization.

The remainder of the paper is structured as follows: the second part is a literature review. The third part shows a mechanical analysis and research hypothesis. The fourth part presents the study design. The fifth part manifests an analysis of the empirical results. The sixth part discusses the mechanism of action, and the seventh part is a conclusion and recommendations.

## Literature review

With the improvement of people’s cognitive abilities and the development of digital technology, the connotation of digital literacy has been deepened and expanded. The concept "digital literacy" was first defined by P. Gilster [11] in his book "Digital Literacy" as people’s ability to access, understand, organize and critique digital information. Subsequent scholars have added to its connotation and extension of digital literacy. Eshet-Alkalai [12] considers digital literacy as the thinking skills to understand and correctly use computers to store digital resources and information. Summey [13] considers digital literacy as the ability of people to have data, digital creation, digital learning, etc. Subsequently, the United Kingdom Joint Information Systems Committee [14] proposed a framework of digital competencies including ICT proficiency, data, and media literacy, digital production and innovation, digital communication and collaboration, digital learning and development, and digital identity and health.Some studies also argue that digital literacy should include digital ethics, engagement, and critical competence [15], clarifying the connotation of digital literacy. In China, Xiao Junhong [16] introduced the term "digital literacy" in 2006, and then "digital literacy" gradually emerged into the vision of domestic scholars. From the perspective of personal competence, Ke Ping [17] pointed out that digital literacy includes the ability to search, read and critically analyze digital information online, and to reorganize knowledge. Based on previous studies, Ling Zhengqiang [18] indicated that digital literacy is a synthesis concept, which is a comprehensive ability of people to use digital tools correctly, retrieve and utilize digital resources critically, and participate in digital social activities freely. In general, along with the continuous penetration of global digital technology, the connotation of digital literacy has progressed from the shallow to the deep, showing dynamic, comprehensive, open, and applied characteristics.

Farmers’ entrepreneurial activities have become a meaningful way to promote rural economic growth and maintain rural economic vitality and are directly related to the process of realizing rural revitalization in China. Regarding the research on the factors influencing farmers’ entrepreneurial behavior, the existing studies can be broadly divided into the following aspects: the first one is individual traits. Farmers will engage in entrepreneurial activities on family farms to survive and succeed, and their entrepreneurial skills significantly impact entrepreneurial activities. One of its main challenges is to enable farmers to develop their entrepreneurial skills, which requires economic support and greater emphasis on education and training [19].Komppula [20], through a case study of Finnish rural tourism, pointed out that a sense of innovation, risk-taking ability, and responsibility are the keys to enhancing the competitiveness of entrepreneurs in rural scenarios.The second is the resource base. Bannor et al. [21] studied Ghanaian farmers involved in producing non-timber forest products using a modified Keynesian test of general entrepreneurial propensity and quantile regression methods. The results presented that most farmers maintained low to average entrepreneurship scores, whereas market information and value-added knowledge positively affected farmers’ entrepreneurship. Díaz-Pichardo et al. [22] researched the transition process of Mexican farmers to entrepreneurs by conducting 28 in-depth interviews. Grande [23] found that traditional folk skills, farm specialties, and peculiar landscape architecture are unique resources that support farmers’ entrepreneurship. The rational exploitation of unique resources and their benefits are the keys to successful entrepreneurship.The last one is the environmental system. Elements such as government control and social norms can significantly influence farmers’ entrepreneurial behavior [24], and an excellent entrepreneurial investment environment and the employment situation of migrant workers can dramatically contribute to farmers’ entrepreneurial behavior [25]. In addition, Fitz-Koch et al. [26] conducted a systematic literature review of existing research in agricultural entrepreneurship, stating that the three critical contextual dimensions of theoretical and empirical directions for future research on entrepreneurship in the agricultural sector are: identity, family, and institution. A dynamic context is provided for scholars’ future entrepreneurship theory and practice research.

A synthesis of the existing literature manifests that there are many research results on farmers’ entrepreneurial behavior, while there needs to be more in-depth research on farmers’ entrepreneurial behavior from the perspective of digital literacy. Based on this, this manuscript may have the following contributions. First, it measures rural people’s digital literacy levels in rural areas of China, deeply analyzes the influence of digital literacy on farmers’ entrepreneurial behavior, extends the study of digital literacy to rural economic activities, and enriches and supplements the research perspective on rural people’s entrepreneurial intentions. Second, it deepens the study of the path mechanism of digital literacy affecting farmers’ entrepreneurial behavior,. As a human capital, health plays an important role in rural economic activities. Analyzing the relationship between digital literacy, health, and farmers’ entrepreneurial behavior and identifying the possible channels through which digital literacy affects farmers’ entrepreneurial behavior, helps to clarify the mechanism path and further exploit the depth of the study. In summary, this paper intends to use the factor analysis method to measure the level of digital literacy based on China Household Finance Survey (CHFS2019) survey data, and investigate the effect of digital literacy on farmers’ entrepreneurial behavior through the Probit model regression and instrumental variables method. Moreover taking health as the entry point, the study of the influence mechanism between digital literacy and farmers’ entrepreneurial behavior was attempted through the mediating effect test. As a result, suggestions are made to improve farmers’ entrepreneurial behavior from the perspective of digital literacy to help rural revitalization, to effectively complement existing studies to a certain extent.

## Empirical findings and hypothesis

### The direct impact of digital literacy on farmers’ entrepreneurial behavior

Information economics theory suggests that prices are obtained at a cost through search and, when received, require further investigation and correction. The relatively high and uneven cost of access leads to an asymmetry in information and competition. Although the information is eternally asymmetric, digital literacy, as "skills and assets in the digital economy," has broken the original temporal and spatial differences and is balancing the information asymmetry to a greater extent, making the information gap between subjects gradually narrowing. Digital literacy is a comprehensive ability that enables farmers to use digital tools, digital technology, digital resources, and digital communication and collaboration with others in a digital context in a proficient, correct, and reasonable manner and to integrate well into digital life and production. It can not only help farmers solve the problems they face in production life by understanding and using digital information and its technology tools, but also help them better integrate into the wave of informatization to obtain resources and advantages in various aspects. Besides, it breaks the information barriers brought by the original geography, distance, and economic development level in rural areas, improves the problem of information asymmetry to a certain extent, and substantially improves their management level. Based on the theory of information economics, this paper argues that the impact of digital literacy on farmers’ entrepreneurial behavior is manifested in the following aspects:

Firstly, digital literacy meets the information needs of farmers’ entrepreneurship. Information is considered a "core resource for entrepreneurial success." Adequate information resources are able to help farmers grasp market dynamics and policy regulations, which in turn help them understand the market and capture business opportunities [27]. In the information era, timely and effective access to relevant information plays a decisive role in entrepreneurial behavior and performance [28]. Influenced by geographical constraints and policy differences, the information obtained in rural areas generally lags, hindering entrepreneurial behavior in rural areas [29]. The fundamental feature of digital literacy is the provision of massive amounts of information to meet users’ ever-changing needs. Improving digital literacy undoubtedly helps expand individual online and offline social networking spaces, and farmers can fully utilize online information resources through digital tools [30] to break information constraints and actively access information concerning entrepreneurship. It helps farmers better grasp market dynamics and relevant policies, making it easier for them to seize entrepreneurial opportunities. For farmer entrepreneurs, the collection and transmission of network information are indispensable, from the capture of business opportunities and expansion of business scope to the opening of sales channels or changes in internal management methods.

Secondly, digital literacy improves farmers’ entrepreneurial capabilities. Digital literacy is essentially a manifestation of competence [12] and can be regarded as a kind of human capital, and farmers with higher digital literacy have higher human capital accumulation. The human capital accumulation of entrepreneurs is the key for improving their entrepreneurship [31], and the higher level of human capital of farmers indicates their higher entrepreneurial ability [32]. Farmers with higher digital literacy can use various digital tools to understand and master the latest technology, providing technical support for their entrepreneurship. Without leaving home, they can quickly know relatively unfamiliar industries and acquire the skills and knowledge needed to start their businesses, which helps to enhance their entrepreneurial capabilities. With the accumulation of knowledge and skills, their entrepreneurial intentions will be stronger. In addition, the advent of the digital economy provides unprecedented opportunities for farmers to start their businesses. They can exploit diverse entrepreneurial approaches starting from e-commerce platforms. The higher the digital literacy of farmers, the better their ability to operate digital platforms such as e-commerce. Moreover, the stronger their digital editing and information dissemination capabilities, and the more efficient they will be in creating output, which will continuously boost farmers’ entrepreneurial confidence and stimulate their entrepreneurial intentions.

Lastly, digital literacy increases the social capital of farmers’ entrepreneurship. Social capital is an explicit and potential resource possessed by individuals, and the more abundant the social capital, the broader the social interaction and the more it can promote individual entrepreneurship [33]. On the one hand, social capital can substantially play the function of social security, significantly reducing the entrepreneurial risks faced by farmers due to insufficient institutional social security and relieving entrepreneurs’ concerns. On the other hand, farmers can access critical resources needed for entrepreneurship by using social capital, such as increasing the possibility of farmers borrowing money from private and banks to alleviate the financial constraints of entrepreneurship [34]. High digital literacy farmers shorten the spatial and temporal distance of mutual interactions through digital tools, drastically reducing the cost of information exchange, breaking spatial limitations, and establishing channels to communicate and connect with their friends [35]. Interpersonal communication in the online platform can be transformed into a real-life social relationship network, promoting social capital accumulation. In addition, leisure activities such as using digital tools to browse social news, sharing on social media, or reading online literature are critical elements in building social capital [36]. Thus, hypothesis 1 is proposed.

**H1**: **Digital literacy has a positive effect on farmers’ entrepreneurial behavior**.

### The mediation effect of health in digital literacy affecting farmers’ entrepreneurial behavior

As a human capital, digital literacy has a significant impact on the health status of rural people. Access to health information can help people actively learn about various types of health knowledge, which can better help them identify health problems and adopt appropriate coping strategies [37]. With the rapid development of the Internet and mobile communication technologies, Internet-based social platforms have become a critical source of health information for people. The more digitally literate farmers are, the better they utilize social platforms such as WeChat, QQ, and Weibo to obtain healthy lifestyle information, which helps them identify diseases and improve their health status. In the era of "digital health", the public is eager to "check" health online and “network self-diagnosis" [38]. The more digitally literate farmers are, the easier to access online health resources, select and utilize online medical care, conduct online medical treatment, and purchase medicines. The utilization of online medical and health resources eliminates information asymmetry between doctors and patients, enhances patients’ understanding of their conditions, and clarifies their medical needs, improving their health status.

Health is an all-around good state of physical, mental, and social relationships [39], which is a prerequisite for all economic activities. As a peculiar occupation, entrepreneurship has prominent characteristics of long working duration, heavy workload, and high work requirements [40], making entrepreneurs more dependent on their health. The health of entrepreneurs affects the identification of entrepreneurial opportunities and the choice of entrepreneurial orientation [41]. When there are health problems, it reduces the ability of individuals to exploit commercial opportunities [42]. At this point, some entrepreneurs may directly shut down their businesses, while others will temporarily cease their operations. However, the business performance will also decrease during the suspension, eventually forcing the entrepreneur to exit the enterprise and they may not generate the idea of entrepreneurship again. Due to the generally low level of education and human capital, for most farmers, maintaining a good physical condition and labor capacity is fundamental for their survival and gaining a foothold. Deteriorating health status will significantly weaken farmers’ employment competitiveness and lead to a continuous decline in their marginal labor remuneration [43]. Improving health status can help promote farmers’ adaptability to the environment, increase their rate of return per unit of time, and enhance the quality of their employment [44]. Farmers who are healthier and more resistant to stress are the ones who choose to be enterprising [45], and accordingly, they are better able to cope with challenges and persist in their entrepreneurial activities. Consequently, hypothesis 2 is formulated.

**H2**: **Health has a mediation effect on digital literacy affecting farmers’ entrepreneurial behavior**

**Fig 1:**
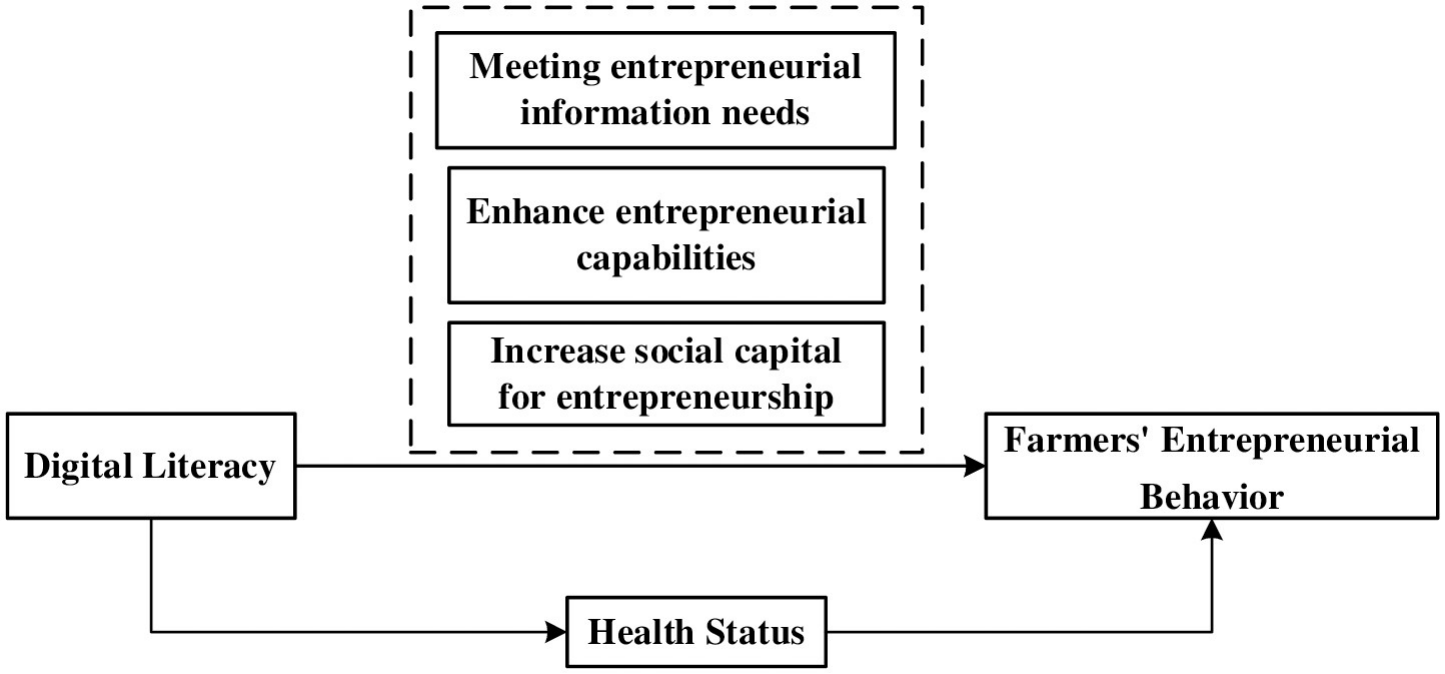
The mechanism of the effect of digital literacy on farmers’ entrepreneurial behavior.

Based on the above analysis, this paper constructs a mechanism diagram on the effect of digital literacy on farmers’ entrepreneurial behavior (see Fig 1).

## Research design

### Data source

Considering the data accessibility and the research topic, this paper adopts the 2019 China Household Finance Survey (CHFS) data published by the China Household Finance Survey and Research Center of Southwest University of Finance and Economics (SWUFE). The CHFS questionnaire covers detailed information on various aspects such as household demographic characteristics, farmers’ entrepreneurship, subjective attitudes, and information about digital literacy, which in turn provides a good data environment for this study. During the data processing, it firstly screened the samples of rural household registration aged 18–80. Then, the main variables, including the relevant variables indicating digital literacy and farmers’ entrepreneurial behavior and the control variables, were selected according to the study objectives and the CHFS questionnaire item settings. Finally, it removed the missing values and outliers of the relevant variables. After the above processing, 25,173 valid samples were obtained in this research.

### Variables selection

(1) Explained variable: Farmers’ entrepreneurial behavior. Farmer entrepreneurial behavior is a market economy activity of farmers with the household as the primary decision-making unit, which mainly refers to the process of value creation by farmers through identifying opportunities, allocating and utilizing resources, and innovating business forms or opening up new business fields [4], including agricultural entrepreneurial activities and non-farm entrepreneurial activities. The entrepreneurial behavior of farmers studied in this paper covers both the behavior of farmers engaging in individual industry and commerce and starting enterprises, as well as the behavior of realizing value through marketization, such as engaging in large-scale or unique breeding and its agricultural products processing. Most of the existing studies on farmers’ entrepreneurial behavior set farmers’ entrepreneurial behavior as a dichotomous variable. Therefore, this paper selects the questionnaire "How many times have you started a business so far? (including self-employment, online store, micro-business, shopping, business enterprise, etc.)" used to measure farmers’ entrepreneurial behavior, and 0 times is defined as no entrepreneurial behavior and assigned a value of 0. In contrast, more than 0 times is given a value of 1.

(2) Core explanatory variables: digital literacy. In this research, five measurement topics were finally selected to measure farmers’ digital literacy at the level of computer use, payment methods, online communication spending, online shopping spending, and online borrowing, as shown in Table 1. Before conducting factor analysis, the KMO test and Bartlett’s sphericity test were conducted on the variable data to determine whether there were common factors among the variables and whether they were suitable for factor analysis. The test results are shown in Table 2. The KMO value of the sample adequacy test is 0.719, which indicates a good correlation between the measurement topics. Meanwhile, the significant P value of Bartlett’s sphericity test statistic is 0, which suggests that the factor analysis results are valid.

Using principal component analysis, two common factors were extracted using the principle that the characteristic root is greater than 1. The cumulative variance contribution of the two factors was 61.45%; the specific results are shown in Table 3. Table 3 shows that common factor 1 can explain 41.43% of the variance and common factor 2 can explain 20.02% of the variance, and the total of the two common factors can explain 61.45% of the variance, which met the requirements. Therefore, a total of 2 common factors were extracted through factor analysis. From the rotated component matrix Table 4, we can see that the 4 variables of whether or not to use a computer, whether or not to pay via cell phone, spending on internet communication, and spending on internet purchases converge on the first common factor. According to the characteristics of these 4 variables, the first common factor can be named as the digital life literacy influence factor. The variables of whether there is Internet borrowing and lending converge in the second common factor, which is named as digital financial literacy factor. The weight of the variance contribution of each factor to the total variance contribution is used as the weight of each factor score to obtain farmers’ digital literacy finally.

(3) Control variables. Li et al. [46] showed that Chinese farmers’ entrepreneurship is mostly family-based, where family and individual characteristics significantly affect entrepreneurship. Therefore, this paper mainly selects control variables at the level of individual and family characteristics. The factors at the individual dimension were selected as gender, age, educational level, and personal well-being because all these factors affect farmers’ entrepreneurial behavior. As the smallest unit of an individual’s life and survival, family is a crucial factor in their behavioral decisions. Thus, factors at the family dimension were chosen as income level, consumption level, health insurance purchase, family size, and whether the family suffered from natural disasters.

(4) Instrumental variables. This paper uses "frequency of online shopping" and "mode of receiving delivery" as instrumental variables for digital literacy. The specific validation and interpretation of the instrumental variables are explained in detail below.

(5) Mediating variable: health status. Firstly, from the subjective perspective, it chooses rural residents’ evaluation of their physical condition to measure health status. Self-rated health satisfaction is adopted to indicate health [39]. Among them, those who considered their health status "very bad" were represented by 1, while those who deemed it "very good" were represented by 5. The scale from 1 to 5 indicates that the self-rated health satisfaction ranges from "the worst" to "the best". Secondly, from the objective perspective, it adopts the annual health care spending to measure the health status of rural residents (spending on tonic health products,blood glucose meters, massage and health instruments, sports equipment, etc.), and generally speaking, the more health care spending, the better their health status.

**Table 1:** Digital literacy assessment questions.

Specific Assessment Questions	Sample Number	Mean Value	Standard Deviation
**Whether to use a computer, yes = 1, no = 0**	25173	0.24	0.43
**Whether to pay via cell phone, yes = 1, no = 0**	25173	0.38	0.49
**Whether there is Internet lending, yes = 1, no = 0**	25173	0.02	0.03
**Online communication spending in 2018 (take natural logarithm)**	25173	4.54	1.22
**Online purchase spending in 2018 (take natural logarithm)**	25173	1.78	3.10

**Table 2:** KMO values and Bartlett’s sphericity test results.

Inspection items and contents		results
**Sampling suitability number (KMO)**		0.719
**Bartlett’s sphericity test**	Approximate cardinality	134.524
	Degree of freedom	78
	Significance (Sig)	0.000

**Table 3:** Total variance explained.

Factors	Eigenvalue	Percentage of variance	Accumulation
**1**	2.07168	0.4143	0.4143
**2**	1.00100	0.2002	0.6145
**3**	0.76724	0.1534	0.7680
**4**	0.67233	0.1345	0.9024
**5**	0.48775	0.0976	1.0000

**Table 4:** Component matrix after rotation.

Variables	Common factor 1	Common factor 2
**Whether to use a computer**	0.6439	
**Whether to pay via cell phone**	0.7804	
**Online communication spending**	0.7910	
**Online purchase spending**	0.6484	
**Whether there is Internet lending**,		0.9967

The definition, assignment and descriptive statistics of each variable are shown in Table 5.

**Table 5:** Variable interpretation and statistical characteristics.

Variable Types	Variable Name	Variable Definition	Sample number	Mean value	Standard deviation
**Explained Variable**	Farmers’ Entrepreneurial Intentions	Willingness of entrepreneurship; Yes = 1, No = 0	25173	0.07	0.25
**Core Explanatory Variables**	Digital Literacy	obtained from factor analysis	25173	-0.05	0.75
**Personal Characteristic Factors**	Gender	Male = 1; Female = 0	25173	0.52	0.50
	Age	From 18 to 80 years old	25173	52.11	16.30
	Educational Level	No schooling = 1; Primary school = 2; Junior high school = 3; Senior high school = 4; Technical secondary school/vocational high school = 5; Junior College/Higher vocational school = 6; Bachelor’s degree = 7; Master’s degree = 8; Doctoral degree = 9	25173	2.73	1.34
	Wellbeing	Very happy = 1; happy = 2; moderately happy = 3; unhappy = 4; very unhappy = 5	25173	2.10	0.89
**Family Characteristics Factors**	Number of Family Members	Actual number of family members	25173	4.14	1.84
	Income Level	Total annual real family income, taking the natural logarithm	25173	14.59	0.13
	Consumption Level	Total annual real family consumption, taking the natural logarithm	25173	11.11	0.53
	Social Health Insurance	Whether purchasing medical insurance. Yes = 1; No = 0	25173	0.93	0.25
	Natural Disasters	Whether family has suffered from a natural disaster in recent years. Yes = 1; No = 0	25173	0.10	0.31
**instrumental variables**	Frequency of online shopping	About how often shopping online. Hardly ever = 1; Sometimes = 2; Often = 3; Always = 4	6112	2.40	0.71
	Express Receiving Method	Whether choosing Cainiao Station to receive express delivery? Yes = 1;No = 0	6421	0.12	0.32
**mediating variable**	Health Status	Self-assessed health status. Very bad = 1; Bad = 2; Average = 3; Good = 4; Very good = 5	25173	3.22	1.06
		Health Care Spending (take the natural logarithm). Actual health care spending in 2018 (spending on tonic health products,blood glucose meters, massage and health instruments, sports equipment, etc.)	25065	0.70	1.94

### Model construction

This empirical model is divided into two parts. First, adopt the binary Probit model to analyze the effect of digital literacy on farmers’ entrepreneurship behavior. Second, adopt the mediating effect model to analyze the logical relationship between digital literacy, health status, and farmers’ entrepreneurial behavior.

1. Probit model. Farmers’ entrepreneurial behavior is a typical binary categorical variable with values of 1 and 0 for the "yes" and "no" options, which is estimated by a binary Probit model [47]. Suppose that farmers’ entrepreneurial intentions are determined by the latent variable *EW*_i_, while *EW*_i_ = *α*_1_*DL*_*i*_ + ∑*X*_*i*_ + *ε*_*i*_, and since *EW*_i_ cannot be observed, all that can be observed is a binary variable. When *EW*_i_ > 0, *EW*_i_ = 1; conversely, when *EW*_i_ > 0, *EW*_i_ = 0. Finally, the specific form of the model to analyze the effect of digital literacy on farmers’ entrepreneurial behavior is as follows.
$$\begin{array}{ll} Probit(EB=1) & =Probit(\gamma_{i}DL_{i}+\sum X_{i}+\varepsilon_{i}>0)\\ & =Probit\left[\varepsilon_{i}>-\left(\gamma_{i}DL_{i}+\sum X_{i}\right)\right]\\ & =1-\varphi \left[-\left(\gamma_{i}DL_{i}+\sum X_{i}\right)\right]=\varphi \left(\gamma_{i}L_{i}+\sum X_{i}\right) \end{array}$$ (1)
  In the model, the explanatory variable *EW* denotes farmers’ entrepreneurial behavior, *EW* = 1 shows farmers with entrepreneurial behavior, and *EB* = 0 presents farmers without entrepreneurial behavior. *φ* manifests the cumulative distribution function of normal distribution, *DL*_*i*_ indicates numerical literacy, and *γ*_*i*_ are coefficients to be estimated for each variable. Besides, *X*_*i*_ denotes control variables affecting farmers’ entrepreneurial behavior at the individual level and household level, and *ε*_*i*_ are random disturbance terms.
2. Mediating effect model. Since the mediating effect model can analyze the influence process and mechanism of the independent variable on the dependent variable and is extensively used in economics research, this paper applies the model to empirically test the mediating role of health status between digital literacy and farmers’ entrepreneurial behavior. In this paper, the stepwise regression method is used to test the mediating effect by referring to Wen et al. [48], which is a more common practice in the existing literature for testing the mediating effect. The approach first tests the influence of the independent variable on the dependent variable; secondly, it examines the impact of the independent variable on the mediating variable; and finally, putting the independent variable, the mediating variable, and the dependent variable in the same model for testing. For this purpose, the specific model settings and steps are as follows.
  Step 1, testing the impact of digital literacy on farmers’ entrepreneurial behavior.
$$EB_{i}=\partial_{1}+\alpha_{1}DL_{i}+\beta_{1}X_{i}+\varepsilon_{i}$$ (2)
  Step 2, testing the impact of digital literacy on health status.
$$Health_{i}=\partial_{2}+\alpha_{2}DL_{i}+\beta_{2}X_{i}+\varepsilon_{i}$$ (3)
  Step 3, integrating digital literacy and health status into the model at the same time.
$$EB_{i}=\partial_{3}+\alpha_{3}DL_{i}+\beta_{3}H{\text{eathi}}_{i}+\beta_{4}X_{i}+\varepsilon_{i}$$ (4)

In the above equation, *EB*_*i*_ denotes farmers’ entrepreneurial behavior, *Health*_*i*_ presents health status, and *DL*_*i*_ indicates digital literacy. Moreover, *∂*_1_, *∂*_2_, *∂*_3_ are constant terms, *β*_*i*_ is the coefficient to be estimated for each variable, *X*_*i*_ represents the control variables affecting farmers’ entrepreneurial behavior at individual and household levels; and *ε*_*i*_ is a random interference term.

The Sobel test and Bootstrap (repeated sampling 5000 times) mediating effect test are further applied respectively. The Sobel method is one of the coefficient product tests, which is different from the stepwise regression method which directly tests the significance of coefficients a and b, without considering the significance of coefficient c. According to the assumption of the Bootstrap method, "confidence interval does not contain zero", that is, when the upper and lower limits of the confidence interval are both positive or negative, it indicates that there is a significant positive or negative effect of mediation.

## Analysis of the empirical results

### Benchmark regression results

This paper conducts a multicollinearity test, and the variance inflation factors are all less than 2, indicating that there is no multicollinearity among the respective variables. To make the results more reliable, this paper adopts a stepwise regression method (see Table 6), and models (1)-(3) are the results of sequentially introducing control variables of farmers’ personal characteristics and family characteristics. The coefficient signs and significance of the results of the three regression equations do not differ significantly, so they are combined for analysis. From the regression results, digital literacy has a positive effect on farmers’ entrepreneurial behavior at the 1% significant level; that is, digital literacy can enhance the behavior of farmers’ entrepreneurship. Thus, hypothesis 1 is confirmed. For other control variables, some factors such as age positively affect farmers’ entrepreneurial behavior at the 1% significant level. The older the farmer is, the more capable he is of accepting new things, and the richer the entrepreneurial capital and social resources he has, the more willing he is to improve his living condition through entrepreneurship. The educational level also positively affects farmers’ entrepreneurial behavior at the 1% significant level. The higher the education level, the stronger the learning ability of farmers, and the more they can learn various skills and increase their chances of success in entrepreneurship, further, their entrepreneurial behavior is getting more and more. Individual happiness positively influences farmers’ willingness to start a business at the 10% significant level. Farmers with a stronger sense of well-being tend to have more energy and a better state for entrepreneurial activities. Meanwhile, entrepreneurial activities generally require some financial support, and farmers with higher incomes tend to have the funds to support entrepreneurship, which promotes their entrepreneurial behavior. The number of family members and natural disasters have a negative effect on farmers’ entrepreneurial behavior at the 5% significant level. It may be because the larger the number of people in a household, the more complex relationships, more tedious things, and higher household expenses. Since entrepreneurship is a risky economic activity that requires much effort, the larger the household size, the more farmers are hesitant to start a business, which inhibits their entrepreneurial behavior. Families suffering from natural disasters often lack enough money and energy for entrepreneurial activities.

**Table 6:** Benchmark regression results.

Variables	(1)	(2)	(3)
**Digital Literacy**	0.3925*** (0. 0146)	0.375*** (0.0153)	0.288*** (0.0168)
**Gender**		-0.0166 (0.0252)	-0.0118 (0.0254)
**Age**		0.00375*** (0.000940)	0.00405*** (0.000986)
**Educational Level**		0.0915*** (0.0105)	0.0796*** (0.0107)
**Happiness**		0.0207 (0.0142)	0.0246* (0.0144)
**Number of Family**			-0.0281*** (0.00785)
**Income level**			1.144*** (0.343)
**Consumption level**			0.333*** (0.0269)
**Social Health Insurance**			0.0718 (0.0544)
**Natural Disaster**			-0.0977** (0.0427)
**_cons**	-1.546*** (0.0129)	-2.036*** (0.0788)	-22.40*** (4.968)
***N***	25173	25,173	25173

Notes:

***p<0.01,

**p<0.05,

*p<0.1;

numbers in parentheses in the table indicate standard errors, the same below.

### Endogenous treatment

This research considers the possible endogeneity of the baseline regression results. On the one hand, digital literacy and farmers’ entrepreneurial behavior may be causally related to each other. Digital literacy affects farmers’ entrepreneurial behavior, and on the contrary, farmers’ entrepreneurial process may be exposed to digital technology and services, and their digital literacy may be higher. On the other hand, there may be omitted variables. Some unobservable factors, such as rural clan power factors, may also affect farmers’ entrepreneurial behavior. As a result, in order to alleviate the endogeneity problem, this manuscript selects "frequency of online shopping" and "whether to choose Cainiao post to receive express delivery" as two instrumental variables of digital literacy in the questionnaire. In addition, it adopts the instrumental variable method to solve the endogeneity problem.On the one hand, the higher frequency of online shopping indicates that farmers are more familiar with all online shopping processes and skilled in using a cell phone or computer shopping APP, all of which will promote the formation of digital literacy. On the other hand, when picking up the express delivery at the Cainiao station, farmers will enter the pickup code or APP to complete the pickup, and all these processes reflect the digital literacy of farmers. Moreover, "frequency of online shopping" and "whether to choose Cainiao post to pick up express delivery" do not directly affect farmers’ entrepreneurial behavior. Therefore, theoretically instrumental variables may be influential instrumental variables of digital literacy.In terms of empirical tests, firstly, the DWH test of whether there is an endogeneity problem was conducted, and the results predicted that the p-value of the DWH test was less than 0.01 and digital literacy was considered as an endogenous variable. Secondly, the minimum eigenvalue statistic of the test is 111.254, which is greater than the critical value of the 10% level test (19.93). Meanwhile, The two instrumental variables are significant at the 1% and 5% levels, respectively, and there is no weak instrumental variable problem. Finally, the p-value in the over-identification test is more significant than 0.1, so the selected instrumental variables can be considered exogenous. In conclusion, it can be assumed that the selected instrumental variables are appropriately and error-free selected(refer to Table 7).

**Table 7:** Estimation results of the instrumental variable method.

变量	First-stage regressions	Second-stage regressions
Digital Literacy		0.336*** (0.041)
Online Shopping Frequency(IV1)	0.167*** (0.012)	
Receive Courier Method(IV2)	0.064** (0.025)	
Minimum Eigenvalue Statistics	111.254	
Control Variables	YES	YES
Sargan Test(P-value		0.2775
Basmann Test(P-value)		0.2779
Sargan (score) chi2		1.1795
Basmann chi2(1)		1.17741
Durbin (score) chi2(1)	53.1918***	
Wu-Hausman F (16100)	53.5534***	
*N*	6112	6112

Based on the above analysis, this paper constructs two-stage least squares (2SLS) to deal with endogeneity using farmer entrepreneurial behavior as the explanatory variable, digital literacy as the core independent variable, and control variables in line with the baseline model. The regression results of the model in Table 7 indicate that after considering the possible endogeneity of digital literacy, the effect of digital literacy on farmers’ entrepreneurial behavior is still significantly positive at the 1% level, which proves that the regression results are relatively robust.

### Robustness test

1. Replacing the explanatory variables: To verify the reliability and robustness of the estimated results of the baseline regression model, the quantitative indicators of the core explanatory variables were replaced by directly selecting the questionnaire "Do you intend to start a business? Yes = 1, No = 0" to redefine farmers’ entrepreneurial behavior. The intention is an essential antecedent variable of behavioral response, directly determining whether and how strongly individual behavior occurs. The stronger an individual’s choice to start a business means that they are more willing to invest more effort in entrepreneurship, thus contributing more to various entrepreneurial activities and exhibiting more entrepreneurial behaviors [49]. In this manuscript, we use farmers’ willingness to start a business to measure entrepreneurial behavior and introduce individual farmer characteristics variables and family characteristics variables to re-run the Probit model regression. As shown in Table 8, the influence coefficient of digital literacy is 0.245, which positively affects farmers’ entrepreneurial behavior at the 1% level. The regression results do not change significantly from the baseline regression, indicating that the regression results are still relatively robust.
2. Replacing the regression model: The previous section of the benchmark regression uses the probit model, and the logit model as a reference to ensure the reliability of the results. The logit model is applicable when the dependent variable is a binary categorical variable or the incidence of an event is a numeric variable. From Table 8, the impact coefficient of digital literacy is 0.564, which still positively affects farmers’ entrepreneurial behavior at the 1% level, and the regression results do not change significantly from the benchmark regression, indicating that they are still relatively robust.

**Table 8:** Robustness test treatments.

Substitution of explanatory variable		Logit model regression	
**Digital Literacy**	0.245*** (0.016)	Digital Literacy	0.564*** (0.039)
**Control Variables**	control	Control Variables	control
**Constant Terms**	-4.440*** (2.260)	Constant Terms	-38.826*** (9.222)
**Observed Values**	25173	Observed Values	25173

### Heterogeneity analysis

To examine the differences of the positive effect of digital literacy on farmers’ entrepreneurial behavior across genders and ages, this paper examines the heterogeneity of digital literacy affecting farmers’ entrepreneurial behavior using respondents’ gender and age as grouping variables, respectively(refer to Table 9). Firstly, it conducts regression analysis by gender, which is divided into male and female samples. The results show that digital literacy in both female and male samples significantly affects farmers’ entrepreneurial willingness at the 1% level, with regression coefficients of 0.278 and 0.299, respectively. It can be seen that women are more visible in using digital literacy as an ability to promote entrepreneurial behavior. The reason may be that compared to men, women have more leisure time to spend on digital tools such as cell phones and computers, and they tend to know more about digital technologies such as mobile internet and big data. Therefore, the positive effect of digital literacy on farmers’ entrepreneurial behavior is larger in the sample of females than that of males. Secondly, according to the actual age of farmers, the samples are categorized into 18~40 years old, 41~64 years old, and 65 years old and above for sub-sample regression. The results show that digital literacy positively affects farmers’ entrepreneurial behavior in the samples aged 18~40, 41~64, and 65 and above at the 1% significant level, with regression coefficients of 0.327, 0.281, and 0.236, respectively. It can be observed that the positive effect of digital literacy on farmers’ entrepreneurial behavior is higher for those aged between 18 and 40. Younger farmers, who are more capable of learning and accepting digital technology, have higher digital literacy, and therefore the promotion of digital literacy on their entrepreneurial behavior tends to be stronger. In contrast, older farmers, affected by their physical condition and cognitive level, tend to be less competent in using digital tools, and thus digital literacy tends to have a lesser impact on their entrepreneurial behavior.

**Table 9:** Regression results by gender and age (Probit regression).

Subsample regression 1			Subsample regression 2		
Variables	Male	Female	18–40 years old	41–64 years old	65 years old and above
**Digital Literacy**	0.278*** (0.023)	0.299*** (0.025)	0.327*** (0.031)	0.281*** (0.024)	0.236*** (0.042)
**Control Variables**	Control	Control	Control	Control	Control
**Constant term**	-23.265*** (6.941)	-21.359*** (1.195)	-27.109*** (8.642)	-22.002*** (7.3001)	-2.180* (1.195)
**Number of samples**	13000	12173	6613	11629	6931

## Further discussions: Analysis of the mechanism of action

### Stepwise regression method

The above results confirm that digital literacy can promote farmers’ entrepreneurial behavior. Regarding the impact mechanism of digital literacy on farmers’ entrepreneurial behavior, this paper takes health status as the entry point and uses the mediating effect model to verify. Table 10 reports the results of the mediating effect test of health status between digital literacy and farmers’ entrepreneurial behavior. According to the results, digital literacy positively affects farmers’ entrepreneurial behavior at the 1% significant level, indicating that the higher the digital literacy, the stronger the farmers’ entrepreneurial behavior. Meanwhile, digital literacy positively affects both self-rated health status and health care expenditure at the 1% significant level. Moreover, digital literacy and self-assessed health status, digital literacy and health care are both included in the model, respectively, resulting that the coefficient of digital literacy, self-assessed health status, and health care expenditures still have positive effects on farmers’ entrepreneurial behavior at the 1% significant level. The above results indicate that health status has a positive partial mediating effect on the influence of digital literacy on farmers’ entrepreneurial behavior. The specific impact is that farmers with higher digital literacy are more able to improve their health level, which in turn promotes their entrepreneurial behavior. Thus hypothesis 2 is confirmed.

**Table 10:** Estimated results of mediating effects of health (OLS).

Procedures	Step 1	Step 2	Step 3	Procedures	Step 1	Step 2	Step 3
Variables	Farmers’ Entrepreneurship Behavior	Self-assessed Health Status	Farmers’ Entrepreneurship Behavior	Variables	Farmers’ Entrepreneurship Behavior	Health Care Spending	Farmers’ Entrepreneurship Behaviors
	0.054*** (0.002)	0.054*** (0.002)	0.054*** (0.003)	Digital Literacy	0.054*** (0.002)	0.271*** (0.019)	0.005*** (0.001)
Self-assessed Health Status			0.004** (0.002)	Healthcare Spending			0.052*** (0.003)
Control variables	Yes	Yes	Yes	Control variables	Yes	Yes	Yes
_cons	-0.713*** (0.175)	3.345*** (00.672)	3.345*** (00.672)	_cons	-0.713*** (0.175)	-7.901*** (1.335)	-0.674*** (0.175)
*N*	25173	25173	25173	*N*	25173	25065	25065

### Sobel and Bootstrap tests

This research further tested the significance of the mediation effect using the Sobel and Bootstrap (5000 replicate sampling) methods(refer to Table 11). First, in the validation of self-rated health status, the Z value of Sobel was 2.239, which passed the 5% significance test, and the confidence interval after the Bootstrap test was [0.046, 0.060], which was significant at the 95% confidence level. Second, in the validation of health care spending, the Z value of Sobel was 5.847, which passed the 1% significance test, and the confidence interval after the Bootstrap test was [0.045, 0.059], which was significant at the 95% confidence level. This proves that health status plays a significant positive mediating effect in the influence of digital literacy on farmers’ entrepreneurial behavior, which again shows the existence of this transmission mechanism. Digital literacy improves farmers’ health, which allows farmers to have more energy and a better state to engage in entrepreneurial activities.

**Table 11:** Results of Sobel and Bootstrap test.

Action Paths	Sobel test (Z value / P value)	Bootstrap (95%) test (Confidence interval)	Test results
Digital Literacy—Self-assessed Health Status—Farmers’ Entrepreneurial Intentions	2.239**	[0.046, 0.060]	Established
Digital Literacy—Healthcare Spending—Farmers’ Entrepreneurial Intentions	5.847***	[0.045, 0.059]	Established

## Conclusions and policy recommendations

In the context of implementing the rural revitalization strategy, farmer entrepreneurship has greatly contributed to farmers’ income and the sustainable development of rural areas. This paper constructs a model of the relationship between digital literacy and farmers’ entrepreneurial behavior. Based on CHFS2019 microdata, it adopts the Probit model and further uses the Ivprobit model with two-stage least squares (2SLS) to deal with endogeneity, systematically examines the influence of digital literacy on farmers’ entrepreneurial behavior, and explores the mediating role of health in the effects of digital literacy on farmers’ entrepreneurial behavior. The study finds that: first, digital literacy is conducive to promoting farmers’ entrepreneurial behavior; second, the positive effect of digital literacy on farmers’ entrepreneurial behavior is more pronounced in the sample of females and the sample of 18 to 40 years old in terms of different genders and ages; third, there is a mediating effect of health in digital literacy in promoting farmers’ entrepreneurial behavior. Previous studies have primarily focused on the relationship between social capital, infrastructure, policies and institutions, and cultural dimensions and the occurrence of entrepreneurship at the national or regional level. Still, few have linked digital literacy to entrepreneurial activities at the micro-individual level. Compared with previous studies, this paper interprets the underlying mechanism of farmers’ entrepreneurship from the digital literacy perspective and considers health status as a mediating variable embedded in the path of "digital literacy influences farmers’ entrepreneurial behavior." Besides, it empirically analyzes the causal relationship between digital literacy first influencing health status and then influencing farmers’ entrepreneurial behavior, which shows the variability of different individual characteristics in digital literacy influencing farmers’ entrepreneurial behavior to cultivate a realistic path for other farmers’ personal digital literacy to achieve optimal entrepreneurship.

According to the above findings, this paper puts forward the following policy recommendations:

First, the level of digital literacy of farmers can be improved. The government and relevant departments can offer training courses to train the skills in using digital tools, digital platforms, etc. Manuals on the use of digital communication tools, digital trading platforms, etc., can be produced, and experts can also visit villages at appropriate times to hold lectures on digital knowledge topics and digital knowledge competition activities. We can arrange for farmers of different genders and ages to be trained in groups and arrange for exceptional people to provide them with targeted training and guidance on using smartphones in an easy-to-understand manner to bring them closer to advanced technologies such as digital technologies. At the same time, according to the personality characteristics and cultural customs of different groups of farmers, some technology-based enterprises should also develop software suitable for different farmers to use so that those groups with limited knowledge can quickly get started with the application. At last, we can provide targeted training for different groups to meet their different entrepreneurial needs through the above methods.
Second, farmers with high digital literacy are encouraged to prioritize entrepreneurial activities. The higher digitally literate farmers are able to start their own businesses through the netroots economy, live-streaming with goods, e-commerce, etc. The government is supposed to encourage these farmers to give priority to entrepreneurial activities so that they can share their new ideas and thinking with neighboring farmers, play a demonstration and leading role, and encourage and guide farmers to establish mutual help entrepreneurial groups. Moreover, it can profoundly broaden the social network system of farmers, promote the smooth flow of digital resources, entrepreneurial information, and capital among farmers, and then give play to the spillover effect of digital literacy so that the driving impact of digital literacy on farmers’ entrepreneurship can be better played.
Third, the government should protect farmers’ "digital health" services. As an essential human capital, health plays a vital role in rural economic activities. In the era of "digital health," on the one hand, farmers should be taught the most practical mobile Internet applications in their daily lives, such as travel and medical treatment, so that they can "check" their health online and "self-diagnose" online through digital platforms, select and use online medical treatment, and conduct online medical treatment and purchase medicines. On the other hand, relevant departments can also provide more convenient and accessible health-intelligent services and promote the construction of health-intelligent service platforms, such as cell phone "intelligent health butler," "Internet + health" fine service platforms, etc., which can effectively meet and protect the health service needs of farmers.

The research in this manuscript has certain limitations that need to be explored in depth. On the one hand, due to the restriction of data, this paper has examined digital literacy in general, and the measurement of digital literacy needs to be further deepened. Further research could be done on digital social literacy, digital security literacy, digital creative literacy, digital business literacy, and so on. On the other hand, this paper only studies farmers’ entrepreneurial behavior, and further analysis of entrepreneurial mode, entrepreneurial types, and entrepreneurial performance can be attempted.
